# Synergistic Structure and Iron‐Vacancy Engineering Realizing High Initial Coulombic Efficiency and Kinetically Accelerated Lithium Storage in Lithium Iron Oxide

**DOI:** 10.1002/advs.202206574

**Published:** 2023-01-22

**Authors:** Naiteng Wu, Jinke Shen, Kai Yong, Chengqian Chen, Jian Li, Yi Xie, Donglei Guo, Guilong Liu, Jin Li, Ang Cao, Xianming Liu, Hongyu Mi, Hao Wu

**Affiliations:** ^1^ Key Laboratory of Function‐oriented Porous Materials of Henan Province College of Chemistry and Chemical Engineering Luoyang Normal University Luoyang Henan 471934 P. R. China; ^2^ State Key Laboratory of Chemistry and Utilization of Carbon Based Energy Resources School of Chemical Engineering and Technology Xinjiang University Urumqi Xinjiang 830046 P. R. China; ^3^ Engineering Research Center of Alternative Energy Materials & Devices Ministry of Education College of Materials Science and Engineering Sichuan University Chengdu Sichuan 610065 P. R. China; ^4^ Department of Physics Technical University of Denmark Lyngby 2800 Denmark

**Keywords:** electronic structure modulation, high initial coulombic efficiency, iron vacancies, lithium iron oxide, lithium‐ion batteries

## Abstract

Transition metal oxides with high capacity still confront the challenges of low initial coulombic efficiency (ICE, generally <70%) and inferior cyclic stability for practical lithium‐storage. Herein, a hollow slender carambola‐like Li_0.43_FeO_1.51_ with Fe vacancies is proposed by a facile reaction of Fe^3+^‐containing metal–organic frameworks with Li_2_CO_3_. Synthesis experiments combined with synchrotron‐radiation X‐ray measurements identify that the hollow structure is caused by Li_2_CO_3_ erosion, while the formation of Fe vacancies is resulted from insufficient lithiation process with reduced Li_2_CO_3_ dosage. The optimized lithium iron oxides exhibit remarkably improved ICE (from 68.24% to 86.78%), high‐rate performance (357 mAh g^−1^ at 5 A g^−1^), and superior cycling stability (884 mAh g^−1^ after 500 cycles at 0.5 A g^−1^). Paring with LiFePO_4_ cathodes, the full‐cells achieve extraordinary cyclic stability with 99.3% retention after 100 cycles. The improved electrochemical performances can be attributed to the synergy of structural characteristics and Fe vacancy engineering. The unique hollow structure alleviates the volume expansion of Li_0.43_FeO_1.51_, while the in situ generated Fe vacancies are powerful for modulating electronic structure with boosted Li^+^ transport rate and catalyze more Li_2_O decomposition to react with Fe in the first charge process, hence enhancing the ICE of lithium iron oxide anode materials.

## Graphical abstract

A hollow slender carambola‐like Li_0.43_FeO_1.51_ with Fe vacancies is constructed via a facile reaction between metal–organic framework (MOF) precursor and Li_2_CO_3_. The in situ generated Fe vacancies are powerful for modulating the electronic structure to accelerate the transport rate of lithium ions and catalyze more decomposition of Li_2_O to participate in the reaction with Fe, finally enhancing the initial coulombic efficiency and rate performance of lithium iron oxide anode material.

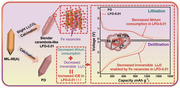

## 1 Introduction

Lithium‐ion batteries (LIBs) have been widely used in energy storage fields because of their high energy density and long cycle life.^[^ ^1^, ^2^ ^]^ With the increasing requirements in electric vehicles, hybrid electric vehicles, and intelligent housing systems, next‐generation LIBs with higher energy density and longer cycle life are required. In the field of anode materials, transition metal oxides with typical conversion reactions enable a high theoretical capacity, which is 2–3 times higher than that of commercial graphite. Among many transition metal oxides, iron‐based oxides have attracted extensive research owing to their high theoretical capacity (1007 mAh g^−1^), high volumetric capacity, low price, and nontoxicity.^[^ ^3^, ^4^ ^]^ It's worth noting that the lithiation process of iron oxide leads to low initial coulombic efficiency (ICE), poor reversibility, and cyclic stability, which would be ascribed to the extra lithium consumption during the formation of solid electrolyte interface (SEI) film and partially irreversible Li_2_O, inferior ion/electron transportation, and unavoidable volumetric expansion, respectively.^[^ ^5^, ^6^ ^]^

Rational design of nanostructures^[^ ^7^, ^8^, ^9^ ^]^ and carbon modification/construction of composite materials^[^ ^10^, ^11^, ^12^ ^]^ are the common strategies to improve the lithium storage performance of iron‐based anodes. Liu et al. designed a carbon‐coated *α*‐Fe_2_O_3_@Fe_3_O_4_ heterostructure, which endowed a reversible capacity of 711 mAh g^−1^ after 200 cycles.^[^ ^13^ ^]^ Heterogeneous mesoporous Mn_2_O_3_/Fe_2_O_3_ showed a high reversible capacity (750 mAh g^−1^ at 1 A g^−1^) and long‐term cyclic life (85.2% after 500 cycles).^[^ ^14^ ^]^ Core–shell structure improved the conductivity and cyclic stability of Fe_2_O_3_/C, which delivered a reversible capacity of 681 mAh g^−1^ after 300 cycles at 1 A g^−1^_._^[^ ^15^ ^]^ Besides, the introduction of lattice defects (mostly oxygen vacancy for metal oxides) by alien or different valence ion doping,^[^ ^16^, ^17^, ^18^ ^]^ purposeful etching,^[^ ^19^, ^20^ ^]^ and designing nonstoichiometric compound,^[^ ^21^, ^22^ ^]^ is another effective route to increase the electronic and ionic conductivity of electrode materials.^[^ ^23^ ^]^ Our previous works had also been encouraged by this strategy to improve the electrochemical performances of TiO_2_,^[^ ^24^, ^25^, ^26^ ^]^ CoO,^[^ ^27^ ^]^ SnO_2_,^[^ ^28^ ^]^ MoO_2_,^[^ ^29^ ^]^ and V_2_O_5_.^[^ ^30^ ^]^ Creation of cationic vacancies is more difficult than that of anionic defects, because of the higher formation energy and complex formation process.^[^ ^31^, ^32^, ^33^ ^]^ However, the above‐mentioned works don't alleviate the additional consumption of lithium during the solid electrode interface (SEI) film formation, resulting in a low ICE (usually lower than 70%). Nowadays, prelithiation is considered as an effective route to increase the ICE of alloying/de‐alloying anode materials. Zhao et al. reported that the ICE of nano‐Si increased from 76% to 94% after partial prelithiation process.^[^ ^34^ ^]^ Such prelithiated route also inspired the research of iron oxide materials. Unfortunately, there are no obvious improvements in ICE, and the ICE of the optimal sample is generally lower than 70%.^[^ ^35^, ^36^ ^]^ LiH has been carried out as a powerful chemical prelithiated reagent to increase the ICE of Si/SiO_x_‐based materials through the high‐temperature solid‐state reaction with their precursors.^[^ ^37^, ^38^ ^]^ Due to its strong reducibility, high price (almost five times than the common lithium salt), and flammability, LiH would be unsuitable for large‐scale applications. Besides, other chemical prelithiation reagents, such as low‐potential Li‐containing chemicals (*n*‐butyl lithium, lithium naphthalene, biphenyl lithium, and lithium‐9,9‐dimethylfluorene)^[^ ^39^, ^40^ ^]^ and Li‐rich additives (Li_x_Si^[^ ^41^ ^]^ and Li_x_Sn^[^ ^42^ ^]^ alloys), also face the problems of oxygen and moisture sensitivity and high cost. Another way to improve the ICE is to decrease the irreversibility of Li_2_O during the first charge process. According to the literatures on electrocatalysis, the cationic vacancies in the electrode materials promoted the decomposition of lithium oxides,^[^ ^43^, ^44^, ^45^ ^]^ which would be carried out to stimulate the reactivity of irreversible Li_2_O. Unfortunately, there are very few reports on the enhancement of ICE from this perspective. Therefore, it remains a significant challenge to seek a green and scalable route to improve the ICE and electrochemical performances of iron oxides.

**Figure 1:**
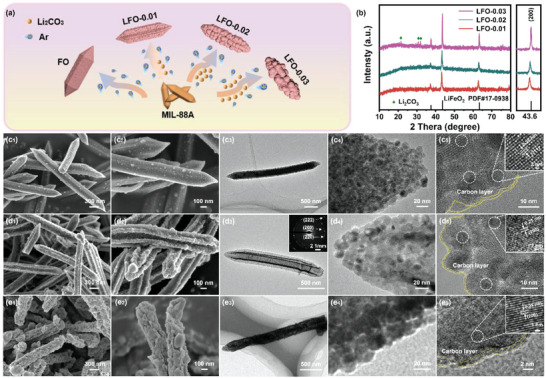
a) Schematic diagram of the preparation processes. b) XRD patterns and SEM, TEM, and HRTEM images of c) FO, d) LFO‐0.01, and e) LFO‐0.02.

In this work, a type of hollow slender carambola‐like lithium iron oxide (Li_0.43_FeO_1.51_, denoted as LFO‐0.01) with Fe vacancies has been synthesized by a facile chemical reaction of MIL‐88A(Fe) with Li_2_CO_3_ at 600 °C under Ar atmosphere (**Figure** **1**a). Metal–organic framework (MOF) precursor not only provides a slender carambola‐like template and porous structure, but also ensures the carbon layer derived from organic groups to increase the conductibility. Interestingly, when a small amount of Li_2_CO_3_ is adopted during the heat‐treatment process, it can simultaneously result in the formation of hollow (caused by lithium erosion) and Fe vacancies (induced by insufficient lithiation process) in the lithium iron oxide. As unveiled by X‐ray absorption fine structure and density functional theory (DFT) calculations, the presence of Fe vacancies is capable of accelerating the transport rate of lithium ions and catalyzing more decomposition of Li_2_O to react with Fe in the first charge process, consequently delivering the improved ICE. When used as anode material for LIBs, the resultant LFO‐0.01 exhibits higher reversible capacity (979 mAh g^−1^ at 0.05A g^−1^), highest ICE (86.78%), best cycling stability (884mAh g^−1^ after 500 cycles at 0.5 A g^−1^), and high‐rate performance than that of the counterparts. Moreover, the LiFePO_4_‖LFO‐0.01 coin full‐cells deliver a high capacity (131 mAh g^−1^, calculated based on the weight of the cathode material) and remarkable cyclic stability of the 99.3% retention after 100 cycles.

## 2 Results and Discussions

The phase compositions of as‐prepared samples were detected through X‐ray diffraction (XRD). As shown in Figure S1a (Supporting Information), after direct pyrolysis of MIL‐88A precursor at 600 °C, the obtained product is standard cubic Fe_3_O_4_ (joint committee on powder diffraction standards, JCPDS No. 65–3107). However, by the addition of Li_2_CO_3_, as‐prepared samples exhibit different diffraction peaks from the blank sample. LFO‐0.01 and LFO‐0.02 show nearly the same XRD patterns assigning to a cubic LiFeO_2_ phase (JCPDS No. 17–0938), except all the diffraction peaks are left shifted compared with the standard pattern (Figure 1b). From the enlarged region, LFO‐0.01 shows a broader and more left‐shifted (200) diffraction peak, indicating the increased lattice parameters than those of LFO‐0.02 and LFO‐0.03.^[^ ^46^ ^]^ In addition, obvious Li_2_CO_3_ impurity can be seen in the LFO‐0.03 pattern, proving that this ratio of lithium and precursor is excessive to form the LiFeO_2_. The Li/Fe ratio of as‐prepared LFO‐0.01 and LFO‐0.02 is determined as 0.43 and 0.87 by Inductively coupled plasma optical emission spectrometer (ICP‐OES), respectively. As shown in Figure S2a (Supporting Information), the weight of MIL‐88A precursor finally remains at 33.51% after the thermogravimetric test, which can be considered as Fe_2_O_3_ formed by the precursor pyrolysis in air. Based on this result, the Fe content of the precursor would be calculated. Combined with the Li/Fe ratio in the preparation process, the molar ratio of Li/Fe in LFO‐0.01 can also be further calculated to be about 0.43, which is consistent with the ICP‐OES results. Thus, the chemical formula of LFO‐0.01 is Li_0.43_FeO_2‐x_. This is a typical nonstoichiometric compound with numerous structural defects, resulting in the left‐shifted diffraction peaks and increased electrons/ions conductivity.

The structures and morphologies of the as‐prepared samples are observed by scanning electron microscope (SEM) and transmission electron microscope (TEM). As shown in Figure S3 (Supporting Information), MIL‐88A precursor displays one‐dimensional sharp cone microrod morphology with about 6–10 µm in length and 300–500 nm in diameter. After direct heat treatment, FO inherits the microrod morphology with a rough and porous surface, which is assembled by countless nanoparticles (Figure 1c_1_,c_2_). TEM image depicted in Figure 1c_3_ further shows the solid rod characterization. The primary particles with a diameter of 15 nm are enclosed with an amorphous carbon layer to tolerate the volume expansion and improve the conductivity (Figure 1c_4_). Moreover, the clear and parallel lattice fringes with an interplanar distance of 0.25 nm are assigned to the (311) facet of cubic Fe_3_O_4_. Remarkably, when slight Li_2_CO_3_ is added and calcined together with the precursor, the rod structure of as‐prepared LFO‐0.01 shrinks inwardly and forms regular cavities, which can be described as slender carambola‐like structure (Figure 1d_1_,d_2_). In Figure 1d_3_, the “keels” supporting a slender carambola‐like structure can be clearly observed. LFO‐0.01 is also assembled by lots of primary particles and coated with carbon layer, in line with the common morphology decomposition from the MOF precursor (Figure 1d_4_). Due to the same precursor utilized, the carbon content of as‐prepared samples could be calculated to about 19.64 wt% according to the weight loss of FO. The diameter of LFO‐0.01 primary particles is similar to the FO counterpart, indicating that the slight addition of lithium salt does not affect their grain size. Furthermore, SAED pattern exhibits the typical polycrystal feature with a series of rings, agreeing with the (200), (220), and (222) crystal plane of crystal defective LiFeO_2_.^[^ ^46^ ^]^ The lattice fringes with a spacing of 0.21 nm belong to the (200) plane of LFO‐0.01. With the increase of lithium carbonate, the morphology of as‐prepared LFO‐0.02 (Figure 1e_1_,e_2_) and LFO‐0.03 (Figure S4, Supporting Information) has been changed gradually. From the TEM images in Figure 1e_3_,e_4_, the voids in the carambola‐like structure have been occupied by the growth primary particles with a diameter of about 25 nm. The increasing grain size of primary particles eventually leads to the collapse of a slender carambola‐like structure. HRTEM image of LFO‐0.02 (Figure 1e_5_) demonstrated that the increased lithium salt addition would not change its interplanar spacing. However, profiles of lattice fringes intensity (Figure S5, Supporting Information) indicate that slight lithium promotes the formation of spot defects, and the number of defects decreases with increasing lithium addition.^[^ ^47^ ^]^ N_2_ adsorption‐desorption isotherms of as‐prepared samples are depicted in Figure S6 (Supporting Information). Brunauer–Emmett–Teller (BET) analyses also verified that the voids in the carambola‐like structure have been occupied. The specific surface area of LFO‐0.02 (95.45 m^2^ g^−1^) and LFO‐0.03 (85.25 m^2^ g^−1^) is obviously lower than that of LFO‐0.01 (116.73 m^2^ g^−1^) and FO (103.07 m^2^ g^−1^). The larger specific surface area can provide more active sites to promote the Li^+^/electrons transfer rate, which would result in the improved rate performance.

**Figure 2:**
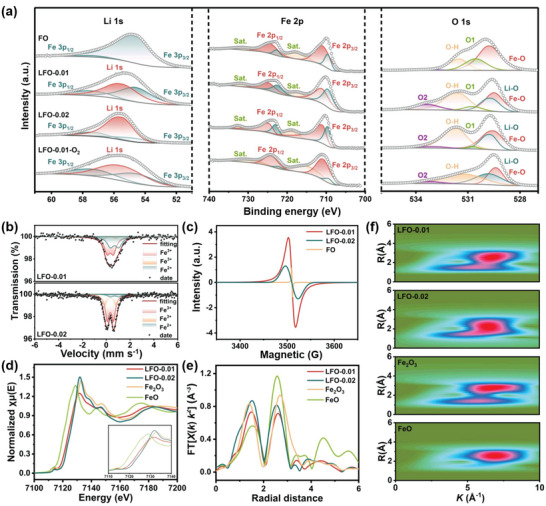
a) XPS spectrums of Li 1s, Fe 2p, and O 1s. b) Mössbauer spectroscopy of LFO‐0.01 and LFO‐0.02; c) EPR results; d) Fe K‐edge XANES spectrums of LFO‐0.01, LFO‐0.02, Fe_2_O_3_, and FeO. e) Fourier transforms of d) Fe K‐edge EXAFS spectra and f) the wavelet transform contour plots at the Fe K‐edge of FO‐0.01, LFO‐0.02, Fe_2_O_3_, and FeO.

X‐ray photoelectron spectroscopy (XPS) was used to investigate the surface element composition and chemical state of as‐prepared samples. As shown in Figure S7 (Supporting Information), all the XPS survey spectra exhibit the similar profile, which verify the presence of Fe, O, and C in the samples. Because of the binding energy Li 1s is close to that of Fe 3p, the peak of Li 1s would be overlapped in the survey spectra. In **Figure** **2**a, the spectrum of FO can be deconvoluted into two peaks, ascribed to Fe 3p_3/2_ and Fe 3p_1/2_, respectively. In the spectrums of LFO‐0.01, LFO‐0.02, and LFO‐0.01‐O_2_, the profiles can be divided into Fe 3p_3/2_, Fe 3p_1/2_, and Li 1s, respectively, demonstrating the presence of Li element. The higher Li 1s peak intensity of LFO‐0.02 means higher content of lithium compared with LFO‐0.01. As revealed by the Fe 2p spectrums, all the samples exhibit a pair of board satellite peaks and two pairs of distinct peaks located at 709.5/722.4 and 710.9/724.6 eV, corresponding to the Fe^2+^ 2p_3/2_/2p_1/2_ and Fe^3+^ 2p_3/2_/2p_1/2_, respectively.^[^ ^48^ ^]^ Owing to the reduction of carbon coating layer, the abundance of Fe^2+^ on the surface of FO, LFO‐0.01, and LFO‐0.02 could induce certain structural defects, which is beneficial to improve the ion/electron conductivity.^[^ ^49^ ^]^ The O 1s spectrum of FO could be fitted as Fe−O bond (529.5 eV), C−O bond (O1, 530.7 eV, detected from the incomplete reduction of carbon coating layer), hydroxyl group (531.5 eV), and absorbed oxygen, respectively. The Fe−O bond, Li−O bond (529.7 eV), C−O bond, hydroxyl group, and absorbed oxygen (O2) can be observed from the LFO‐0.01 and LFO‐0.002 spectrums. According to Figure S1b (Supporting Information), LFO‐0.01‐O_2_ is a mixture of LiFeO_2_ and Fe_2_O_3_. The spectrum of LFO‐0.01‐O_2_ displays four peaks belonging to the Fe‐O bond, Li‐O bond, hydroxyl group, and absorbed O_2_. The carbon coating layer was oxidized during the heat‐treatment in air. Moreover, the high ratio of hydroxyl peak means a strong coupling between cationic vacancies and the hydroxyl species.^[^ ^32^ ^]^ In terms of the charge balance principle, the Fe^3+^/Fe^2+^ ratio is an important prerequisite for the verification of *x* value in LFO‐0.01. XPS cannot reflect the intrinsic valence state of materials because of its limited detection depth. Mössbauer spectroscopy has been considered as a powerful tool to estimate the valence state, coordination number of chemical bonds, crystal structure, electron density, and magnetic properties.^[^ ^50^ ^]^ As shown in Figure 2b, Mössbauer spectrums of LFO‐0.01 and 0.02 exhibit obviously different profiles, owing to the different lithium salt addition. Both LFO‐0.01 and LFO‐0.02 Mössbauer spectroscopy could be fitted as three doublet peaks with an isomer shift of 0.3379, 0.3426, and 0.7028 mm s^−1^, which belonged to the valence site of Fe^3+^, Fe^3+^, and Fe^2+^, respectively.^[^ ^50^ ^]^ The detailed site parameters of two samples are listed in Table S1 (Supporting Information). After fitting the area of split peaks, the Fe^3+^/Fe^2+^ ratio of LFO‐0.01 can be calculated as about 1.44. Further combined with the Li/Fe ratio, the *x* value can be estimated as 0.49. The chemical formula of LFO‐0.01 would be determined to Li_0.43_FeO_1.51_. Similarly, the chemical formula of LFO‐0.02 is identified as Li_0.87_FeO_1.89_.

Electron paramagnetic resonance (EPR) is a physical method to detect the unpaired electron in materials with defects/vacancies by the *g* value under various chemical environments. The strong symmetrical EPR peaks of FO, LFO‐0.01, and LFO‐0.02 with a *g* value of 2.003, 2.001, and 2.001 are observed in Figure 2c, respectively, which meant the existence of electrons captured by structural vacancies. The *g* value of 2.003 is evidence of oxygen vacancies, meaning that LFO‐0.01 and LFO‐0.02 possessed the different type of vacancies from the FO counterpart.^[^ ^32^ ^]^ It's worth noting that the intensity of ERP peak corresponds to the concentration of unpaired electrons.^[^ ^29^ ^]^ LFO‐0.01 delivers the highest defect concentration which would benefit the electrons/ions transport. To further analyze the structure of LFO‐0.01 and LFO‐0.02, the Fe K‐edge X‐ray absorption near edge structure (XANES) and Fourier‐transformed extended X‐ray absorption fine structure (FT‐EXAFS) were carried out. The K‐edge absorption energy of LFO‐0.01 and LFO‐0.02 lines are between that of FeO and Fe_2_O_3_ (Figure 2d). From the enlarged absorption edges, the average iron valence of LFO‐0.02 is higher than that of LFO‐0.01, implying more Fe^3+^ content coupled with the more lithium salt addition. The FT‐EXAFS spectrums of LFO‐0.01 and LFO‐0.02 depicted in Figure 2e present the typical Fe−O bond with one chief peak located at 1.47 Å, one shoulder peak (Fe−Fe bond) at 2.62 Å.^[^ ^51^ ^]^ The intensity of peaks in *R* space reflects the coordination relationship of the corresponding bonds.^[^ ^52^, ^53^ ^]^ According to the minimum intensity of shoulder peak, the coordination number of Fe in LFO‐0.01 is reduced, which would infer that the defect type in LFO‐0.01 is Fe vacancies.^[^ ^54^ ^]^ Additionally, the enlarged wavelet transform EXAFS maximum profile indicated that LFO‐0.01 delivered more diverse coordination environments than the counterparts due to the Fe vacancies,^[^ ^49^, ^55^ ^]^ which would improve its electrochemical performances. In the process of calcination to form LiFeO_2_, the reduced Fe^2+^ by carbon layer would tend to occupy the Li^+^ sites in the case of insufficient lithium addition, which induced cation vacancies compensation to maintain the electrical neutrality.^[^ ^56^ ^]^ This tendency decreases with the increase of Li_2_CO_3_ addition.

**Figure 3:**
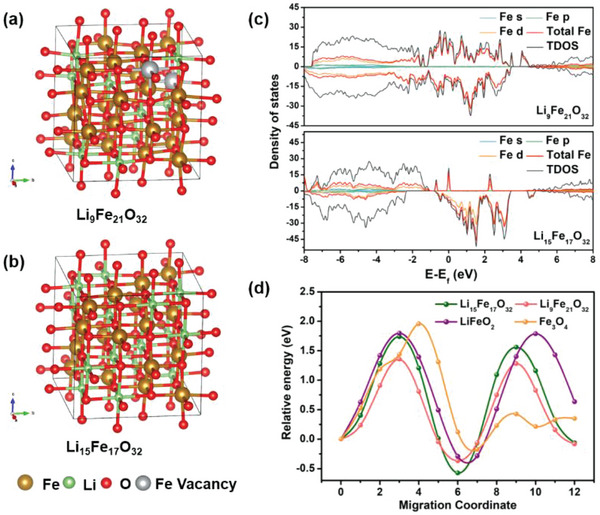
The structural models of a) Li_9_Fe_21_O_32_ and b) Li_15_Fe_17_O_32_. c) Density of state of two models of Fe. d) Migration energy profiles of Li^+^ in Li_9_Fe_21_O_32_, Li_15_Fe_17_O_32_, LiFeO_2_, and Fe_3_O_4_.

Density functional theory (DFT) calculations were conducted to understand the structural features of LFO‐0.01 with Fe vacancies. A 2 × 2 × 2 Li_16_Fe_16_O_32_ supercell structure containing 64 atoms was constructed. To generate Fe^2+^, the numbers of lithium and iron atoms are purposefully adjusted in the unit cell to balance the valence and keep the cubic structure. However, their positions are still randomly generated. Based on the practical atom ratio of Li/Fe and the content ratio of Fe^3+^/Fe^2+^, the geometrically optimized models of LFO‐0.01 and LFO‐0.02 would be approximated to Li_9_Fe_21_O_32_ and Li_15_Fe_17_O_32_, respectively (**Figure** **3**a,b). There are two Fe vacancies in the Li_9_Fe_21_O_32_ supercell structure. The calculated total density of states (TDOS) and partial density of states (DOS) of Fe are depicted in Figure 3c. By the creation of Fe vacancies, continuous and obvious states of Fe d orbital near the Fermi level could be observed, which let transportation of electrons much easier than that of Li_15_Fe_17_O_32_.^[^ ^25^, ^46^ ^]^ Besides, the DOS of Li and O further demonstrate that Fe is a key role in the variation of models’ DOS near the Fermi level (Figure S8, Supporting Information). Moreover, the lithium‐ion migration energy barrier in Li_9_Fe_21_O_32_ is only 1.35 eV, which is much smaller than those of Li_15_Fe_17_O_32_ (1.74 eV), LiFeO_2_ (1.80 eV), and Fe_3_O_4_ (1.96 eV), further demonstrating more easily Li^+^ migration process in Li_9_Fe_21_O_32_ unit cells. Due to the DFT calculation results, the presence of Fe vacancies in the structure would not only modulate the electronic structure to improve electrical conductivity of LFO‐0.01, but also promote the Li^+^ diffusion to accelerate charge storage processes simultaneously.

**Figure 4:**
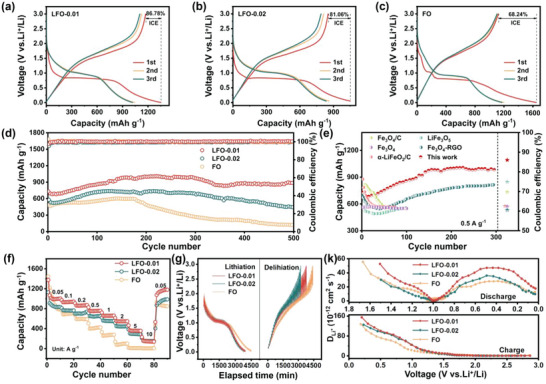
Initial three discharge–charge curves of a) LFO‐0.01, b) LFO‐0.02, and c) FO. d) Long‐term cyclic performances under the current density of 0.5 A g^−1^, f) rate performances, g) GITT curves and k) Li^+^ diffusion coefficient at different states of LFO‐0.01, LFO‐0.02, and FO. e) Comparison of cyclic performance and initial coulombic efficiency (ICE) between LFO‐0.01 and reported Fe‐based anodes.

The electrochemical performances of as‐prepared samples were measured by CR2032 half‐coin cells. As shown in **Figure** **4**a, LFO‐0.01 delivers the first discharge and charge capacity of 1352 and 1173 mAh g^−1^, respectively, with an ICE of 86.78%. As the counterpart, LFO‐0.02 (Figure 4b) synthesized with double the amount of Li_2_CO_3_ exhibits a lower capacity (1053/853 mAh g^−1^) and ICE (81.06%), indicating that increasing the amount of Li_2_CO_3_ would limit its electrochemical activity. However, both LFO‐0.01 and LFO‐0.02 possess the higher ICE than that of FO (68.24%, Figure 4c) electrode. Overlapped discharge–charge curves demonstrate all of the as‐prepared samples have good lithium intercalation/deintercalation reversibility. Moreover, initial cyclic voltammetry (CV) curves of as‐prepared electrodes at the scan rate of 0.1 mV s^−1^ in the voltage range of 0.005‐3 V are depicted in Figure S9 (Supporting Information), which accorded with the typical lithiation/delithiation behavior of Fe‐based anode materials. During the first discharge, the obvious cathodic peak located at 0.6 V is the reduction of Fe^3+^/Fe^2+^ and the formation of Li_2_O. In the anodic scan, a broad peak at around 1.68 V can be assigned to the delithiation and the Fe^0^ oxidation.^[^ ^4^, ^11^, ^46^ ^]^ The well‐overlapped cathodic and anodic peaks in the next three cycles mean their excellent reversible lithium storage performance.

Figure 4d depicts the cycling performance and coulombic efficiency of as‐prepared samples at a current density of 0.5 A g^−1^. Impressively, the LFO‐0.01 electrode remained the discharge specific capacity of 884 mAh g^−1^ after 500 cycles, which is much higher than that of LFO‐0.02 (445 mAh g^−1^), LFO‐0.03 (203 mAh g^−1^, Figure S10a, Supporting Information) and FO (118 mAh g^−1^) electrodes. The d*Q*/d*V* curves of LFO‐0.01 at different cycles in Figure S11 (Supporting Information) show the similar profiles, meaning its high reversibility of lithium storage. Moreover, the electrochemical performances of LFO‐0.01 are obviously superior to the reported Fe‐based anode materials in cycling capacity and ICE (Figure 4e).^[^ ^57^, ^58^, ^59^, ^60^, ^61^ ^]^ As shown in Figure 4f and Figure S10b (Supporting Information), LFO‐0.01, LFO‐0.02, and LFO‐0.03 exhibit the improved rate performances at high current density, which is attributed to their increased Li^+^/electrons diffusion rates. The LFO‐0.01 electrode shows the best rate performance, and delivers the specific capacity of 1000, 930, 860, 753, 658, 545, 357, 145, and 1148 mAh g^−1^ at 0.05, 0.1, 0.2, 0.5, 1, 2, 5, 10, and back to 0.05 A g^−1^ respectively. However, the diffusion contribution ratio of LFO‐0.01 is larger than that of FO, meaning the improved diffusion kinetics of LFO‐0.01 (Figure S12, Supporting Information). This interesting phenomenon is reversed to the reported modified iron oxide anodes.^[^ ^62^, ^63^ ^]^ The increased diffusion contribution ratio of LFO‐0.01 would be attributed to the modulated charge distribution by the presence of Fe vacancies, and consequently weakened the electrostatic interaction to promote Li^+^ diffusion in the LFO‐0.01 structure.^[^ ^64^ ^]^ Galvanostatic intermittent titration technique (GITT) method is carried out to estimate the lithium‐ion diffusion coefficient of as‐prepared samples. As shown in Figure 4g, all the GITT curves profiles are corresponding to their discharge–charge curves. The lithium diffusion coefficient (*D*_Li_) of three electrodes can be calculated according to the following Equation (1) (the meaning of parameters is supplemented in Figure S13, Supporting Information).^[^ ^25^ ^]^ During the discharge–charge periods (Figure 4k), all of the electrodes display the same variation trends. And the LFO‐0.01 owns the highest *D*_Li_ to promote the rate performance.

(1) $$D_{\mathrm{GITT}}=\frac{4}{\pi \tau }{\left(\frac{m_{B}V_{M}}{M_{B}S}\right)}^{2}{\left(\frac{\Delta E_{s}}{\Delta E_{t}}\right)}^{2}$$

Furthermore, electrochemical impedance spectroscopy (EIS) is used to evaluate the resistance of charge transfer (*R*_ct_) at different cycles. All the Nyquist plots (Figure S14, Supporting Information) deliver two semicircles and a line in the middle and high‐frequency regions at 100^th^ cycle, representing the surface film resistance (*R*_f_), *R*_ct_, and Warburg impedance (*Z*_w_), respectively.^[^ ^65^ ^]^ Compared with the counterparts (the *R*_ct_ of FO and LFO‐0.02 is 2046 and 192.5 Ω, respectively), the smallest *R*_ct_ of LFO‐0.01 at 100^th^ cycle (156.6 Ω) would be attributed to the presence of Fe vacancies, which promoted the charge transfer processes. With the increase of cycles, the impedances of the three electrodes exhibit similar decreasing trends, implying the continuous improvement in charge storage kinetics. GITT and EIS analyses clearly demonstrate that the existence of Fe vacancies in LFO‐0.01 facilitates ion diffusion and charge transfer during the charge–discharge processes, which is consistent with the DFT calculation results. Besides, the LFO‐0.01 and FO batteries after 50 cycles at 0.5 A g^−1^ have been dissected and characterized by SEM to check the morphology evolution. Evidently, LFO‐0.01 still retains the slender carambola‐like morphology with the thin SEI film on the surface (Figure S15a,b, Supporting Information). As the counterpart, FO microrods display the enlarged diameter, rough surface, and pulverized particles (Figure S15d,e, Supporting Information), which proved that the structural stability of LFO‐0.01 is better than that of FO. The SEM images of the electrode cross profile depicted in Figure S15c (Supporting Information) show that the thickness increase of LFO‐0.01 electrode is only about 40%, far less than that of FO (increased by120%, Figure S15f, Supporting Information). According to the above results, the impressive improvement of LFO‐0.01 would be attributed to the slender carambola‐like structure and the Fe vacancies induced by the non‐stoichiometric design. These structural features not only alleviate the volume expansion and accelerate ion/electron diffusion rates during the monotonous cycles, but also reduce the irreversible capacity loss and endow more active sites to obtain the high reversible capacity.

**Figure 5:**
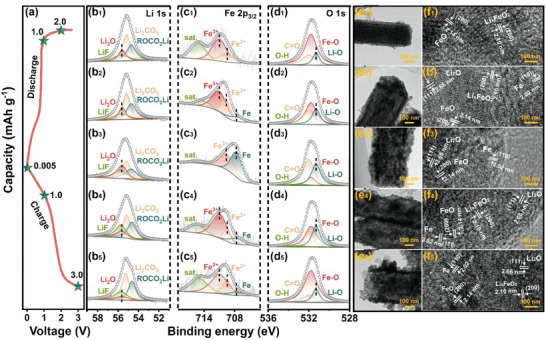
a) Initial charge–discharge curve, b) Li 1s spectrums, c) Fe 2p_3/2_ spectrums, d) O 1s spectrums. e) TEM images and f) HRTEM images of LFO‐0.01 at different states.

Ex situ XPS and TEM are performed at different discharged–charged states in the Li^+^ half‐cells to explain the lithium storage mechanism. After first discharged to 2 V, the Li 1s spectrum (**Figure** **5**b_1_) could be divided into the ROCO_2_Li, Li_2_CO_3_, Li_2_O, and LiF peaks, representing the formation of SEI film and the reduction of iron ions. Limited by the detection depth of XPS, the weak signals of Fe element have been collected, which were affected by the formed SEI film (Figure 5c). Thus, the interference of Fe 3p signal in Li 1s can be ignored by the low intensity of Fe 2p signal. The relative content of Li_2_O reaches the maximum at 0.005 V, and gradually decreases at the charge stage. At the end of the first charge (Figure 5b_5_), the residual signal of Li_2_O is mainly attributed to the SEI film and irreversible Li_2_O. Fe^0^ signal intensity is getting higher with the increasing discharge depth. At 0.005 V (Figure 5c_3_), an obvious Fe^2+^ peak still exists, implying the incomplete reduction processes. During the charging process, the content of Fe decreases continuously, and a slight Fe^0^ has found at a cut‐off voltage of 3 V (Figure 5c_5_), indicating that the charging process was also incomplete, and foreshadowed for the subsequent capacity rising. At the following cycles, these active materials would be gradually exposed to enhance the lithium storage ability. In O 1s spectrums (Figure 5d), the change tendency of Fe−O and Li−O bonds are consistent with analyses of ex situ Li 1s and Fe 2p spectrums. As shown in Figure 5e, the one‐dimensional morphology of LFO‐0.01 can be clearly observed at different states. Furthermore, at the first discharged to 2 V, (200) plane of FeO and (200) plane of Li_x_FeO_2_ is observed in the HRTEM image (Figure 5f_1_). The (110) plane of Fe can be found in the following discharged at 1 V (Figure 5f_2_). Li_x_FeO_2_ is absent at discharged of 0.005 V, and reappears at the charged of 1 and 3 V, implying the conversion reaction between Li_x_FeO_2_ and FeO.

**Figure 6:**
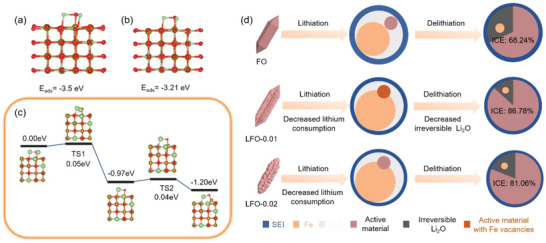
Optimized structure of Li_2_O adsorbed on the (010) plane of a) Li_9_Fe_21_O_32_ and b) Li_15_Fe_17_O_32_. c) The Gibbs free energy diagram of Li_2_O cluster decomposition on the surface of Li_9_Fe_21_O_32_ during the first charge process. d) Schematic diagram of initial coulombic efficiency (ICE) improvement processes.

Moreover, the anode material had not been reduced completely in the first discharge process, according to the ex situ XPS and TEM analyses. The unreduced LFO‐0.01 may play a critical role in the charging process. To verify this conjecture, DFT calculations using the above models are carried out. **Figure** **6**a,b displays the optimized structure of Li_2_O absorbed on the (010) plane of Li_9_Fe_21_O_32_ and Li_15_Fe_17_O_32_, respectively. The adsorption energy (*E*_ads_) calculation results show that the *E*_ads_ on Li_9_Fe_21_O_32_ surface (the model of LFO‐0.01) is significantly lower than that of Li_15_Fe_17_O_32_ (the model of LFO‐0.02), inferring that the presence of Fe vacancies acts as the active site to promote the capture of Li_2_O. This result may be the critical factor for the higher ICE of LFO‐0.01 than LFO‐0.02. Figure 6c depicts the Gibbs free energy diagram of Li_2_O cluster decomposition and formation of Li_x_FeO_y_ on the surface of Li_9_Fe_21_O_32_ during the first charge process. From the calculated energy diagram, the reaction between Li_2_O and Fe on the surface of Li_9_Fe_21_O_32_ (presence of the Fe vacancies) to form Li_x_FeO_y_ goes through five steps. After the first step of Li_2_O adsorption, Δ*G* of transient state (TS) 1 and TS2 are only 0.05 and 0.04 eV, respectively. Besides, the Δ*G* of the whole reaction is −1.20 eV, indicating the thermodynamically spontaneous reaction. DFT calculations indicate that Fe vacancies, as active sites, would capture Li_2_O, catalyze the reaction between Fe and Li_2_O, and reduce the amount of irreversible Li_2_O simultaneously, which is regarded as the main factor to the ICE improvement. Hence, the ICE improvement processes can be illustrated in Figure 6d. In the first lithiation process, LFO‐0.01 reduced the lithium consumption compared with FO, which is reflected in the lower first irreversible discharge capacity. At the subsequent charge process, decreasing the amount of irreversible Li_2_O and catalyzing more Li_2_O to participate in the following reaction with Fe can directly increase the charge capacity, and improve the ICE of LFO‐0.01 consequently.

**Figure 7:**
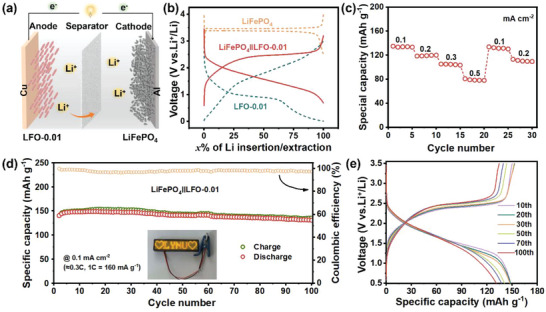
a) Schematic illustration of the full‐cell configuration. b) Typical charge–discharge curves of LiFePO_4_‖LFO‐0.01 full‐cell. The c) rate performance, d) cyclic performance, and e) charge–discharge curves at different cycles of LiFePO_4_‖LFO‐0.01 full‐cell.

Furthermore, the LiFePO_4_‖LFO‐0.01 coin full‐cells are assembled by using the commercial LiFePO_4_ as cathode with the negative/positive capacity (N/P) ratio of 1.2 (**Figure** **7**a), encouraged by the outstanding electrochemical performances of LFO‐0.01 electrode in a half‐cell. The electrochemical performances of commercial LiFePO_4_ in half‐cells are exhibited in Figure S16 (Supporting Information). As shown in Figure 7b, the voltage window of the LiFePO_4_‖LFO‐0.01 full‐cell is set between 0.5 and 3.5 V. The capacity of the full‐cell is calculated based on the weight of the cathode material. In the evaluation of rate performance (Figure. 7c), LiFePO_4_‖LFO‐0.01 full‐cell delivers a specific capacity of 134, 120, 105, 80, 133, and 113 mAh g^−1^ at the current density of 0.1, 0.2, 0.3, 0.5, back to 0.1, and 0.2 mA cm^−2^, respectively, which indicates the good rate capability of LiFePO_4_‖LFO‐0.01 full‐cell. At the following cyclic test (Figure 7d), LiFePO_4_‖LFO‐0.01 full‐cell shows superior cyclic stability with a high capacity of 131 mAh g^−1^, 99.2% capacity retention and close to 100% coulombic efficiency after 100 cycles at 0.1 mA cm^−2^ (≈0.3 C, 1 C = 160 mA g^−1^). The inset in Figure 7d is a digital photo of “♡LYNU♡” LEDs device lighted by a coin full‐cell. Besides, the monotonous charge–discharge plateaus at different cycles (Figure 7e) demonstrate that LiFePO_4_‖LFO‐0.01 full‐cell would deliver a stable voltage for electrical equipment.

## 3 Conclusion

In summary, a type of lithium iron oxide with hollow slender carambola‐like structure and Fe vacancies has been synthesized by the thermal reaction between MIL‐88A precursor and Li_2_CO_3_. The structure features and electrochemical performances of as‐synthesized lithium iron oxides have been obviously affected by the addition of lithium salt. A small amount of Li_2_CO_3_ leads to the formation of hollow LFO‐0.01 with Fe vacancies during the heat‐treatment process. DFT calculations show that the presence of Fe vacancies accelerates the transport rate of lithium ions and catalyzes more decomposition of Li_2_O to participate in the reaction with Fe. Thus, LFO‐0.01 delivers the superior performances in lithium storage, including the high ICE (86.78%) and stable cyclic property (884 mAh g^−1^ after 500 cycles at 0.5 A g^−1^). The LiFePO_4_‖LFO‐0.01 coin full‐cells also exhibit the high capacity (131 mAh g^−1^) and stable cyclic performance (99.3% retention after 100 cycles). This work would encourage other transition metal oxides with conversion‐type lithium storage mechanism to improve their electrochemical performances.

## Conflict of Interest

The authors declare no conflict of interest.

## Supporting information

Supporting Information

## Data Availability

Research data are not shared.
