# Supplementary material for: Synergistic Structure and Iron‐Vacancy Engineering Realizing High Initial Coulombic Efficiency and Kinetically Accelerated Lithium Storage in Lithium Iron Oxide
Source: Adv Sci (Weinh). 2023 Jan 22;10(9):2206574. doi: 10.1002/advs.202206574 (PMC10037985; doi:10.1002/advs.202206574)
Supplement: Supplementary file 1 — Supporting Information [file ADVS-10-2206574-s001.pdf]

Supporting Information

Synergistic Structure and Iron-Vacancy Engineering Realizing High Initial Coulombic Efficiency and Kinetically Accelerated Lithium Storage in Lithium Iron Oxide

Naiteng Wu, Jinke Shen, Kai Yong, Chengqian Chen, Jian Li, Yi Xie, Donglei Guo, Guilong Liu, Jin Li, Ang Cao, Xianming Liu, Hongyu Mi*, Hao Wu**

Prof. N. Wu, J. Shen, C. Chen, J. Li, Y. Xie, Dr. D. Guo, Dr. G. Liu, Dr. J. Li, Prof. X. Liu
Key Laboratory of Function-oriented Porous Materials of Henan Province, College of Chemistry and Chemical Engineering
Luoyang Normal University
Luoyang, Henan, 471934, China
E-mail: myclxm@163.com
J. Shen, Prof. H. Mi
State Key Laboratory of Chemistry and Utilization of Carbon Based Energy Resources, School of Chemical Engineering and Technology
Xinjiang University
Urumqi, Xinjiang, 830046, China
E-mail: mmihongyu@163.com
K. Yong, Prof. H. Wu
Engineering Research Center of Alternative Energy Materials & Devices, Ministry of Education, College of Materials Science and Engineering
Sichuan University
Chengdu, Sichuan, 610065, China
E-mail: hao.wu@scu.edu.cn
Dr. A. Cao
Department of Physics
Technical University of Denmark
Lyngby, 2800, Denmark

Section SI. Experimental Section

Chemicals and materials

All of the used reagents in this work are analytical grade and obtained from Aladdin (Shanghai, China).

Synthesis of MIL-88A

Typically, 0.139 g fumaric acid was dissolved in 25 mL deionized water under magnetic stirring at 70 °C. Then, 0.525 g $Fe(NO_3)\cdot 9H_2O$ was added to the above solution with continuous stirring for 10 min. Subsequently, the mixture was transferred into a 50 mL Teflon-lined stainless-steel autoclave and heated at 110 °C at 6 h. After cooling to room temperature, the orange-yellow solution was filtered and washed with water and ethanol, respectively. Finally, the as-prepared precipitate was dried in air at 60 °C for 12 h.

Synthesis of slender carambola-like lithium iron oxide

For the preparation of $Li_{0.43}FeO_{2-x}$ (denoted as LFO-0.01), 0.01 g Li_2CO_3 and 0.15 g MIL-88A precursor (the mass ratio of Li_2CO_3: MIL-88A = 1: 15) was mixed uniformly in a mortar, the obtained mixture was heated to 600 °C for 3 h under Ar atmosphere.

Synthesis of the counterparts

For comparison, LFO-0.02 and LFO-0.03 were prepared through same processes except 0.02 and 0.03 g of Li_2CO_3 (the mass ratio of Li_2CO_3: MIL-88A = 1: 7.5 and 1: 5) were added, respectively. Besides, mixed 0.01 g Li_2CO_3 and 0.15 g MIL-88A precursor also calcined at 600 °C for 3 h under air atmosphere to remove carbon layer and oxygen vacancies (denoted as LFO-0.01-O_2). For the preparation of Fe_3O_4, the MIL-88A precursor was sintered at 600°C for 3 h under Ar atmosphere and denoted as FO.

Theoretical calculation details

All Spin polarization density functional theory (DFT) calculations were performed in generalized gradient approximation (GGA) with the Perdew-Burke-Ernzerhof (PBE) formulation.[S1-S3] Projected augmented wave (PAW) potentials have been used to describe the

ionic cores and take valence electrons into account using a plane wave basis set with a kinetic energy cutoff of 400 eV. [S4, S5] Partial occupancies of the Kohn−Sham orbitals were allowed using the Gaussian smearing method and a width of 0.05 eV. The electronic energy was considered as self-consistent, when the energy change was less than 10^{-5} eV. A geometry optimization was considered convergently when the energy change was smaller than 0.05 eV $Å^{-1}$. The vacuum spacing in a direction perpendicular to the plane of the structure is 15 Å. The Brillouin zone integration is performed using 3×3×3 Gamma k-point sampling for a structure. Finally, the adsorption energies (E_{ads}) were calculated as equation (1), where $E_{ad/sub}$, E_{ad}, and E_{sub} are the total energies of the optimized adsorbate/substrate system, the adsorbate in the structure, and the clean substrate, respectively.

$$E_{ads} = E_{ad/sub} - E_{ad} - E_{sub} \quad \text{(Eq. 1)}$$

The free energy was calculated using the following equation (2), where G, E, ZPE and TS are the free energy, total energy from DFT calcu lations, zero point energy and entropic contributions, respectively. In our calculation, the U correction had been set as 5.3 eV for Fe atoms in our systems.

$$G = E + ZPE - TS \quad \text{(Eq. 2)}$$

Materials Characterizations

The X-ray diffraction (XRD) analysis was performed on a Bruker D8 with Cu Kα radiation to determine the crystal structure of the samples. The morphologies and structures of the as-prepared samples were characterized by scanning electron microscope (SEM, Zeiss Sigma 500) and transmission electron microscope (TEM, FEI Talos F200S). X-ray photoelectron spectroscopy (XPS, Thermo Scientific K-Alpha) was carried out to determine valence states of the samples. The thermal gravimetric analysis was performed on a thermal analyzer (SII TG/DTA6300) in air. The specific surface areas of the samples were evaluated by Brunauer-Emmett-Teller method (BET, ASAP 2020 Plus HD88). The room temperature ^{57}Fe Mössbauer spectra were recorded using a proportional counter and a Topologic MFD-500AV-

02 spectrometer with ^{57}Co (Rh) as a γ-ray radioactive source. The velocity was calibrated by a standard α-iron foil. Electron paramagnetic resonances (EPR) were carried out on a Bruker EMXPlus-10/12 spectrometer. X-Ray absorption fine structure spectroscopy (XAFS) measurements were performed at the 21A X-ray nanodiffraction beamline of Taiwan Photon Source.

Electrochemical measurements

The working electrode was manufactures by dispersing active material, carbon black (conductive agent) and polyvinylidene fluoride (PVDF, binder) in N-methyl-2-pyrrolidone (NMP) solvent at a mass ratio of 7:2:1 to form a uniform slurry. Then, the slurry was coated on the copper foil substrate and dried at 100 °C in a vacuum for 12 h. The electrode cut into discs with a diameter of 1.2 cm were assembled into CR-2032 coin-type batteries in a glove box filled with argon for electrochemical performance test. The electrolyte was composed of a mixture of ethylene carbonate (EC), dimethyl carbonate (DMC) and diethyl carbonate (DEC) (volume 1:1:1). The galvanostatic charge/discharge were measured on a Neware apparatus (Shen Zhen, CT-3008W). The electrochemical impedance spectra (EIS) of the cells were carried out in the frequency range of 100 KHz to 0.01 Hz at an AC amplitude of 5 mV on a Parstat 4000+ electrochemical workstation. Cyclic voltammetry (CV) measurements were also performed on the Parstat 4000+ workstation in the potential range from 0.005 to 3.0 V (vs. Li^+/Li) with different scanning rates. During the typical Galvanostatic Intermittent Titration Technique (GITT) measurement, the battery was charged and discharged with a pulse time (τ) of 20 min under a 50 mA g^{-1} pulse current density, then followed by a relaxation time of 60 min.

The commercial $LiFePO_4$ electrodes were used instead of metallic lithium to assemble the cion full-cells. The $LiFePO_4$ electrodes were prepared by mixing 70% of active material, 10% carbon black and 10% PVDF in NMP solution under stirring to form slurry. The slurry was coated on the aluminum foil substrate and dried at 100 °C in a vacuum for 12 h. The

LFO-0.01 electrode was chemical prelithiation for 3 cycles at the current density of 100 mA g^{-1} before the fabrication of full-cell. The capacity of LFO-0.01 electrode and $LiFePO_4$ electrode is controlled approximately to be 1.2:1. The electrolyte was composed of a mixture of ethylene carbonate (EC), dimethyl carbonate (DMC) and diethyl carbonate (DEC) (volume 1:1:1). The galvanostatic charge/discharge (GCD) tests were performed in the voltage ranges of 0.5-3.5 V.

Section SII. Supporting Figures and Table

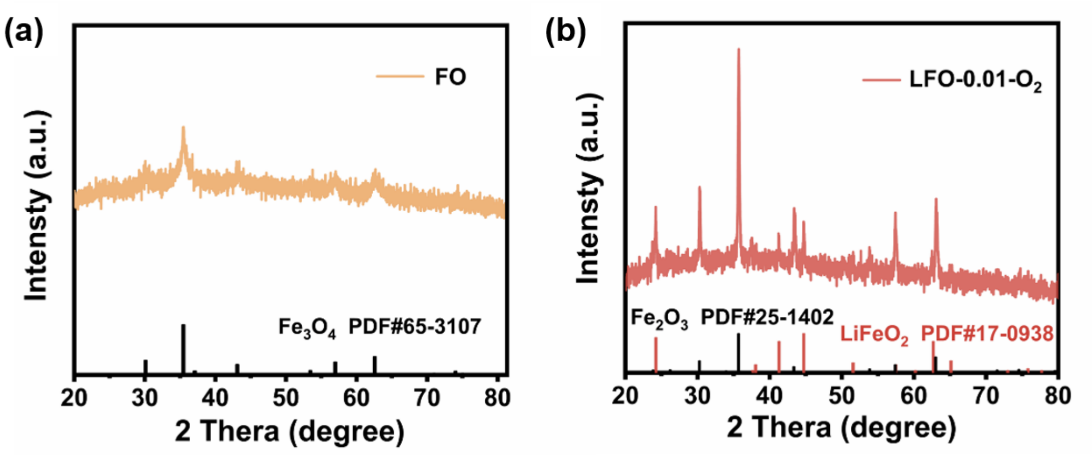

Figure S1 XRD patterns of (a) FO and (b) LFO-0.01-O_2

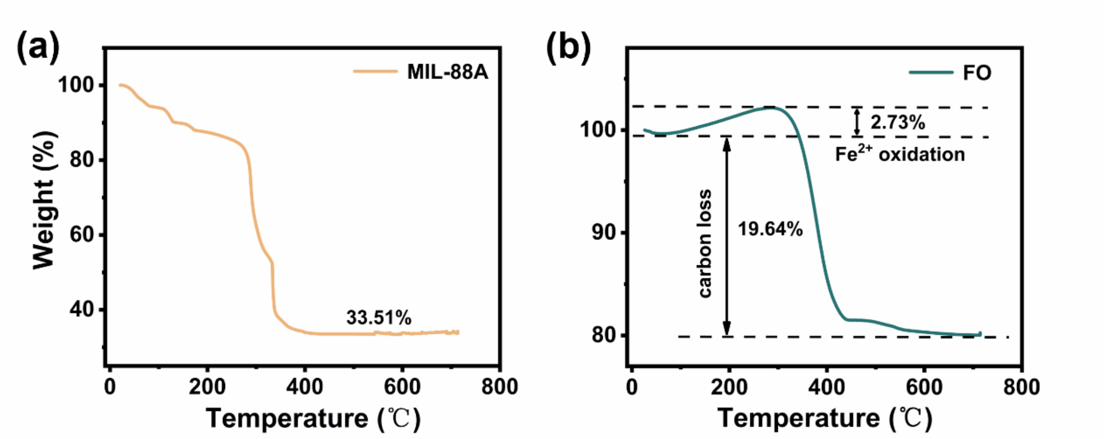

Figure S2 TG curves of MIL-88A percusor and FO in air

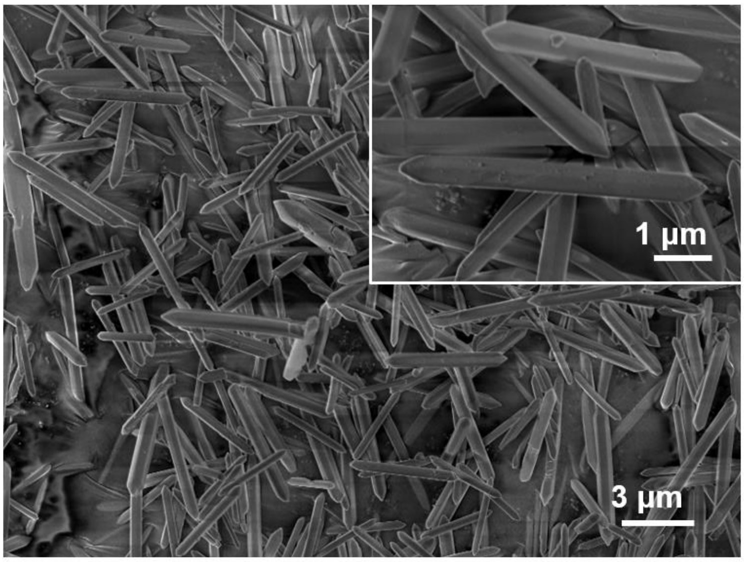

Figure S3 SEM images of MIL-88A precursor

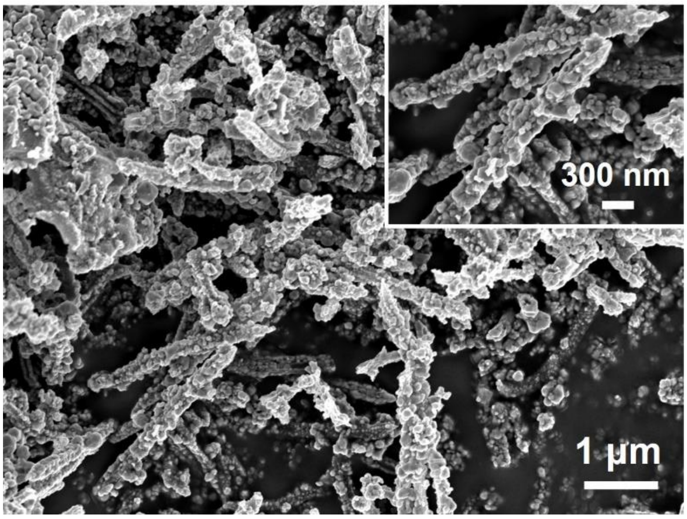

Figure S4 SEM images of LFO-0.03

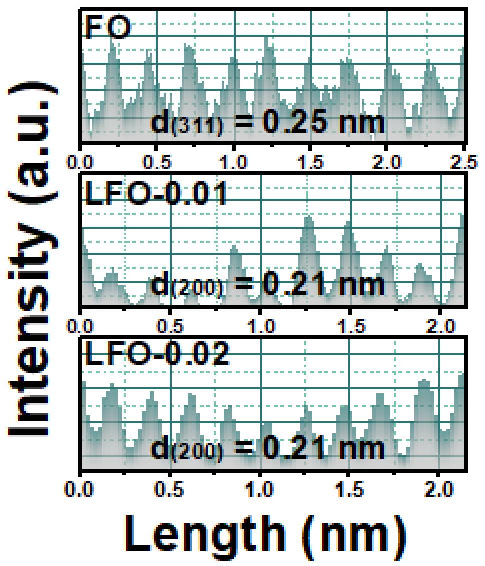

Figure S5 The profile plots with a line of lattice fringes intensity

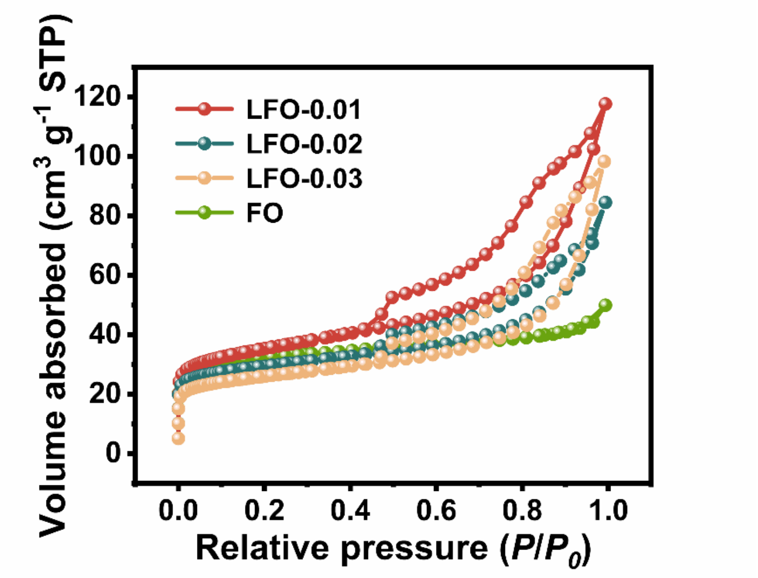

Figure S6 N_2 adsorption-desorption isotherms of as-prepared samples

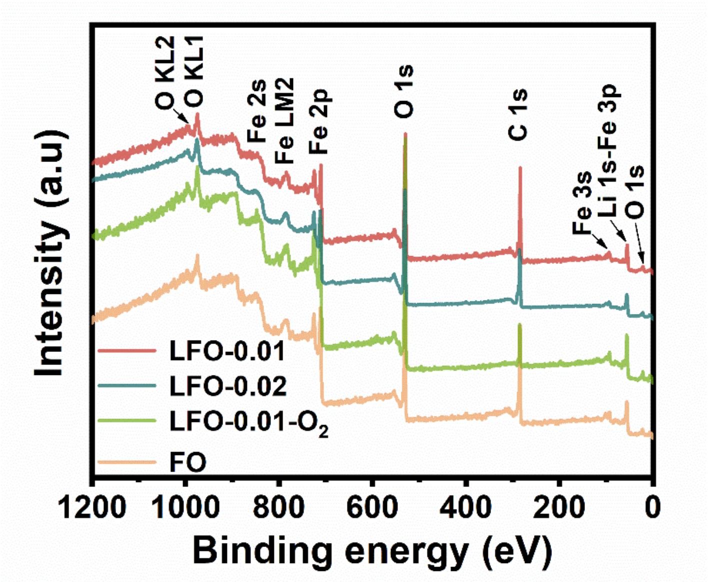

Figure S7 XPS survey spectra of as-prepared samples

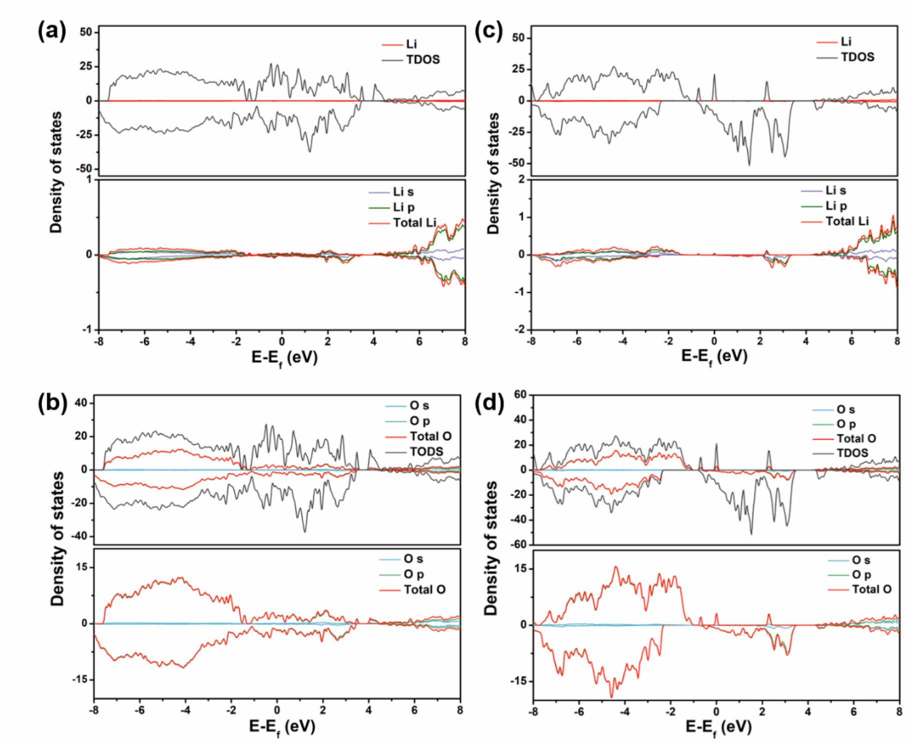

Figure S8 Density of states of Li and O of (a and b) $Li_9Fe_{21}O_{32}$ and (c and d) $Li_{15}Fe_{17}O_{32}$

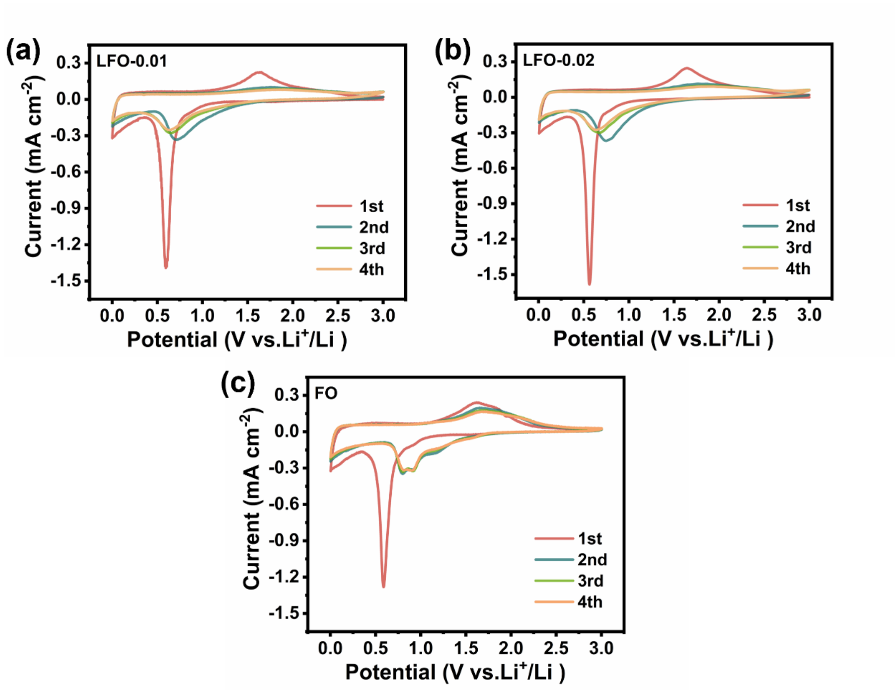

Figure S9 CV curves of (a) LFO-0.01, (b)LFO-0.02 and (c) FO

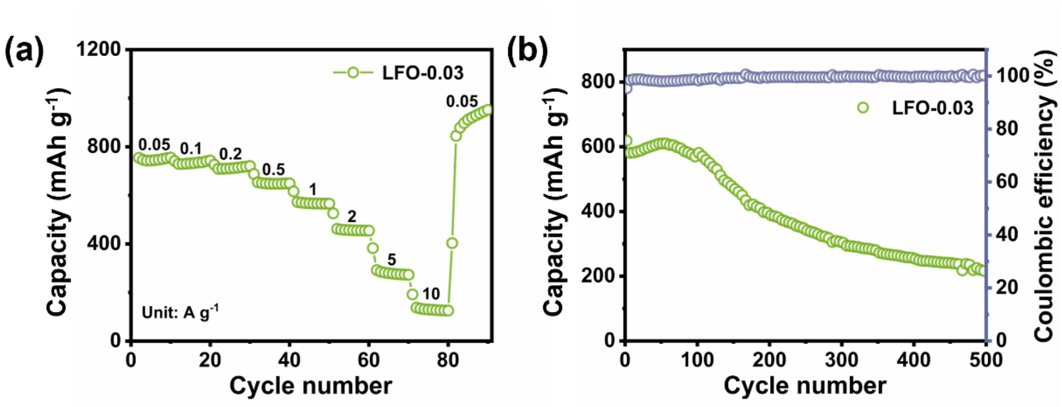

Figure S10 (a) Cycle and (b) rate performances of LFO-0.03

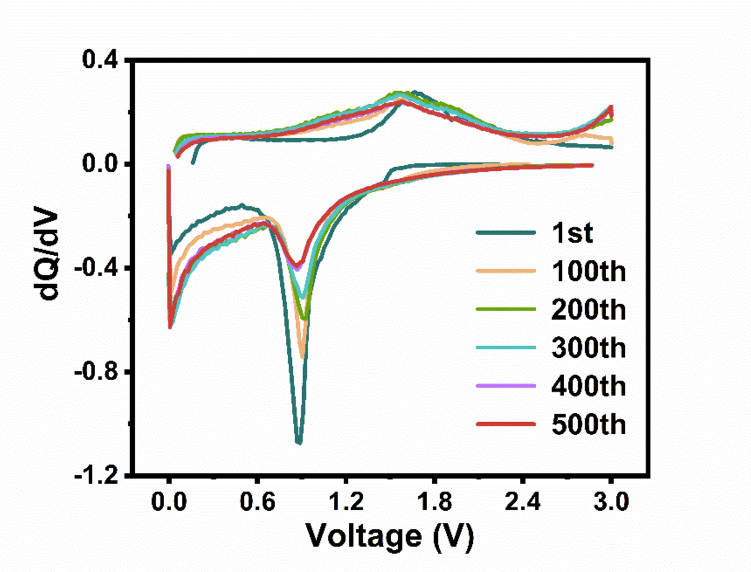

Figure S11 dQ/dV curves of LFO-0.01 at different cycles

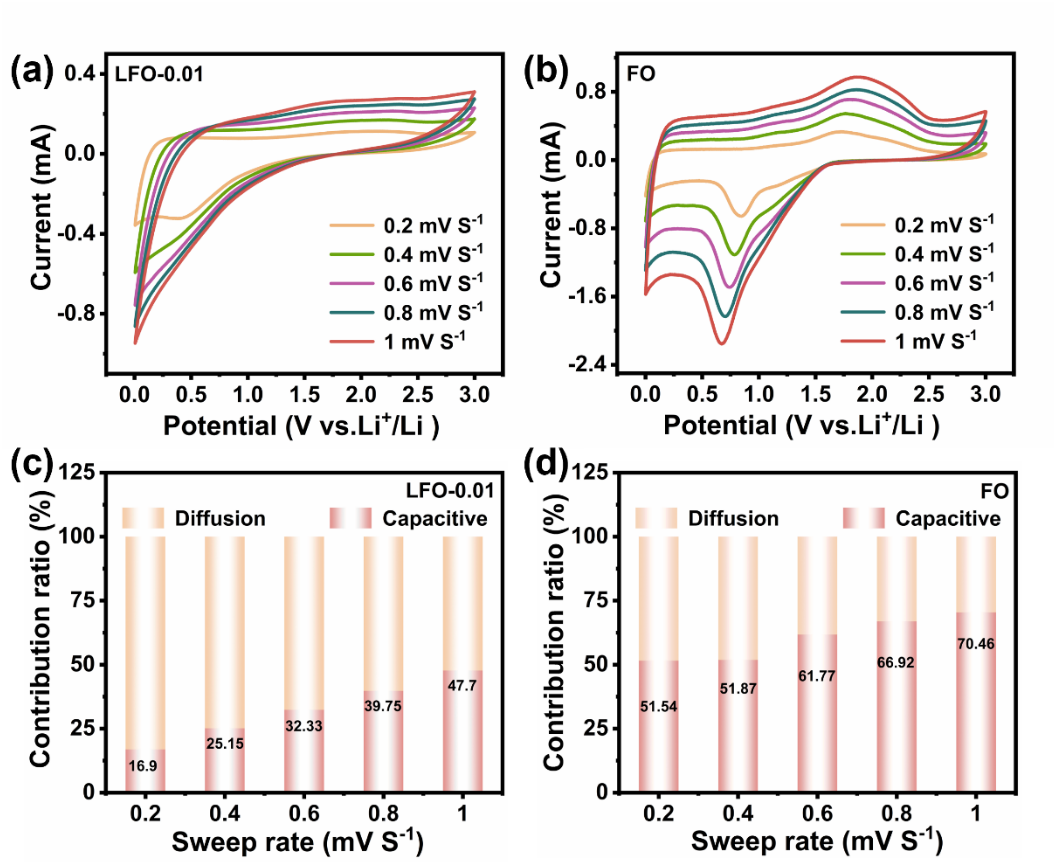

Figure S12 Electrochemical kinetics: CV curves and capacitive and diffusion contribution ratios at different scanning rates of (a and c) LFO-0.01 and (b and d) FO

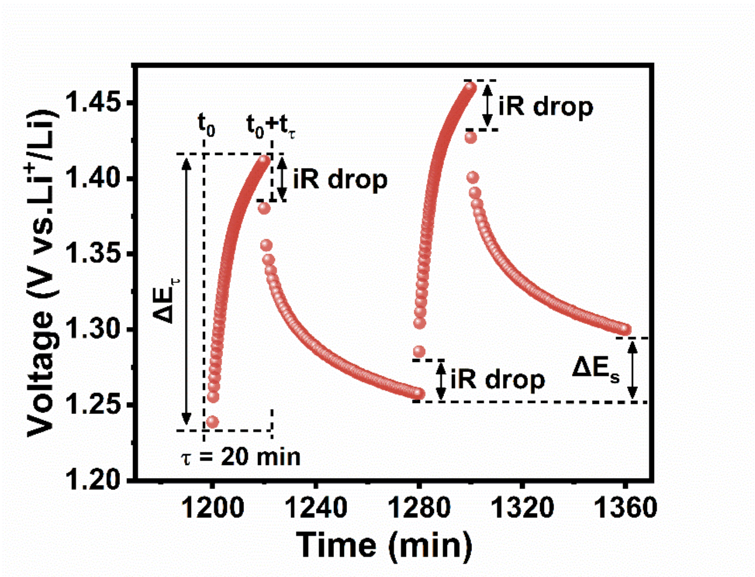

Figure S13 Enlarge GITT curves of LFO-0.01 at charge process

τ, *m*, *M*, V_m and *S* in the equation are the current pulse time, mass, molar mass, molar volume and electrode surface area of electrode material, respectively. ΔE_τ is the change of battery voltage during the pulse, and ΔE_s is the voltage drop between the initial and steady state.

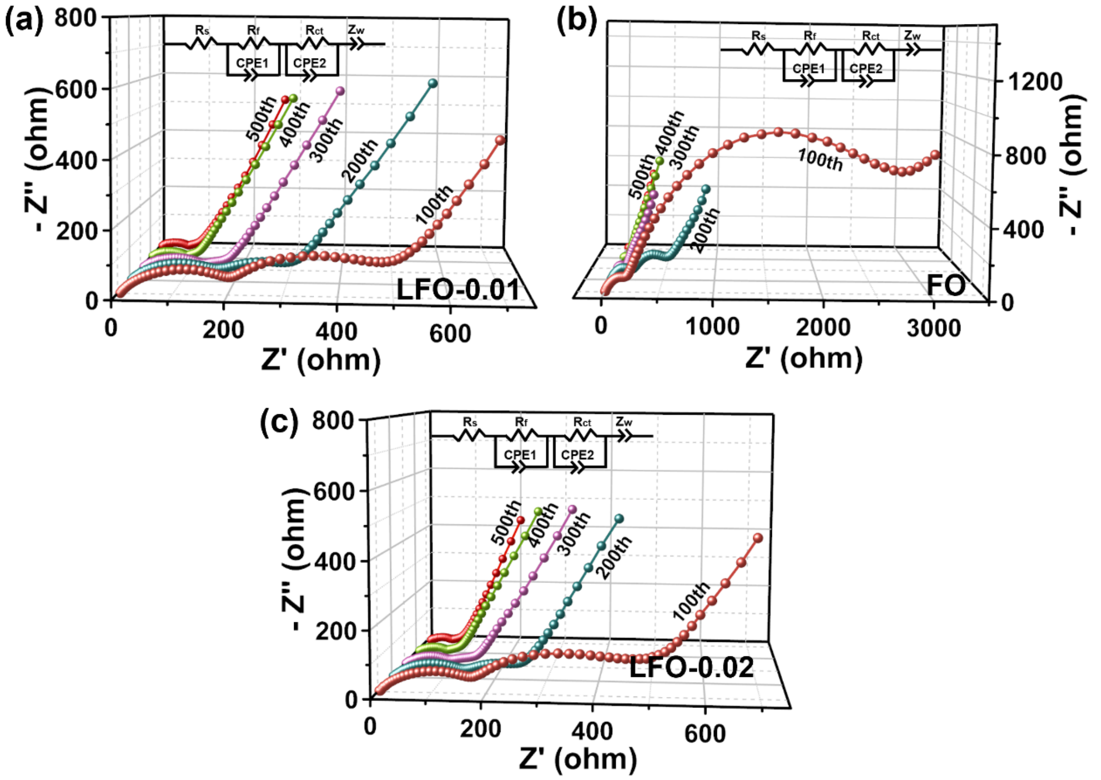

Figure S14 Nyquist plots of (a)LFO-0.01, (b) FO and (c) LFO-0.02 at different cycles

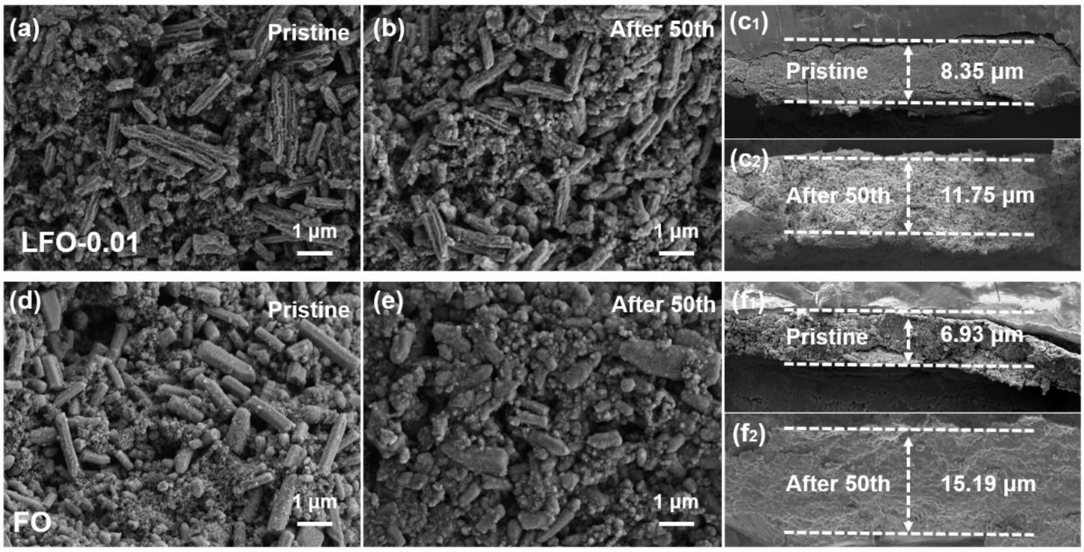

Figure S15 SEM images of pristine and cycled electrodes of (a-c) LFO-0.01 and (d-f) FO

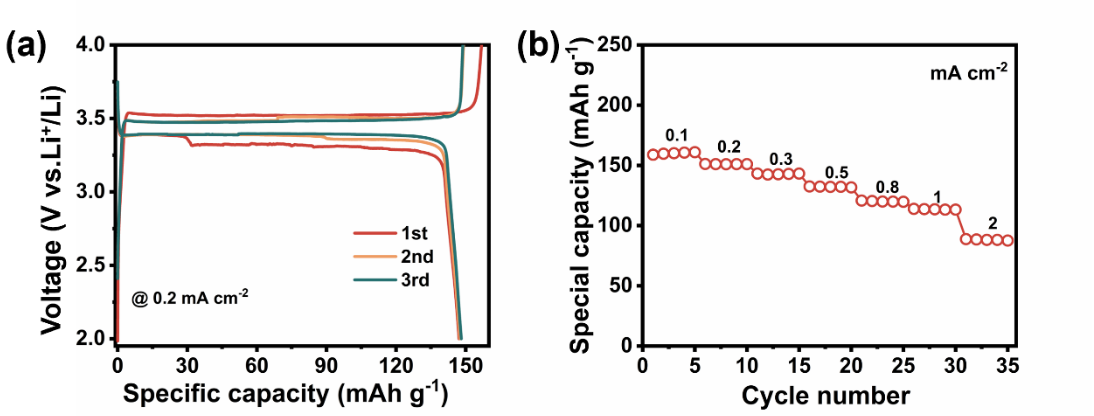

Figure S16 (a) The initial charge-discharge curves of commercial $LiFePO_4$ at 0.2 mA cm^{-2}. (b) The rate performances of commercial $LiFePO_4$

Table S1 The site parameters obtained from fitting the ^{57}Fe Mössbauer spectrums of LFO-0.01 and LFO-0.02 at room temperature: isomer shift IS, quadrupole coupling QS, line width, and area fraction.

Sample	Peak Shape	IS (mm/s)	QS (mm/s)	Line width	Area fraction	Affiliation
**LFO-0.01**	Doublet 1	0.3379	0.9383	0.3654	6.11%	Fe^{3+}
	Doublet 2	0.3426	0.5232	0.5557	52.84%	Fe^{3+}
	Doublet 3	0.7028	0.7102	0.6556	41.05%	Fe^{2+}
**LFO-0.02**	Doublet 1	0.3379	0.9383	0.4049	28.93%	Fe^{3+}
	Doublet 2	0.3426	0.5232	0.4049	62.75%	Fe^{3+}
	Doublet 3	0.7028	0.7102	0.5797	8.32%	Fe^{2+}

References

[S1] G. Kresse, J. Furthmüller, *Comput. Mater. Sci.* **1996**, *6*, 15.

[S2] G. Kresse, J. Furthmüller, *Phys. Rev. B* **1996**, *54*, 11169.

[S3] J. P. Perdew, K. Burke, M. Ernzerhof, *Phys. Rev. Lett.* **1996**, *77*, 3865.

[S4] G. Kresse, D. Joubert, *Phys. Rev. B* **1999**, *59*, 1758.

[S5] P. E. Blöchl, *Phys. Rev. B* **1994**, *50*, 17953−17979.
